# Insight into the Effect of Inhibitor Resistant S130G Mutant on Physico-Chemical Properties of SHV Type Beta-Lactamase: A Molecular Dynamics Study

**DOI:** 10.1371/journal.pone.0112456

**Published:** 2014-12-05

**Authors:** Mohd Hassan Baig, D. Raja Sudhakar, Ponnusamy Kalaiarasan, Naidu Subbarao, Gulshan Wadhawa, Mohtashim Lohani, M Kalim A Khan, Asad U. Khan

**Affiliations:** 1 Interdisciplinary Biotechnology Unit, Aligarh Muslim University, Aligarh, India; 2 Department of Biosciences, Integral University, Lucknow, India; 3 School of Computational and Integrative Sciences, Jawaharlal Nehru University, New Delhi, India; 4 National Centre of Applied Human Genetics, School of Life Sciences, Jawaharlal Nehru University, New Delhi, India; 5 Department of Biotechnology, Government of India, New Delhi, India; NCBS-TIFR, India

## Abstract

Bacterial resistance is a serious threat to human health. The production of β-lactamase, which inactivates β-lactams is most common cause of resistance to the β-lactam antibiotics. The Class A enzymes are most frequently encountered among the four β-lactamases in the clinic isolates. Mutations in class A β-lactamases play a crucial role in substrate and inhibitor specificity. SHV and TEM type are known to be most common class A β-lactamases. In the present study, we have analyzed the effect of inhibitor resistant S130G point mutation of SHV type Class-A β-lactamase using molecular dynamics and other *in silico* approaches. Our study involved the use of different *in silico* methods to investigate the affect of S130G point mutation on the major physico-chemical properties of SHV type class A β-lactamase. We have used molecular dynamics approach to compare the dynamic behaviour of native and S130G mutant form of SHV β-lactamase by analyzing different properties like root mean square deviation (RMSD), H-bond, Radius of gyration (Rg) and RMS fluctuation of mutation. The results clearly suggest notable loss in the stability of S130G mutant that may further lead to decrease in substrate specificity of SHV. Molecular docking further indicates that S130G mutation decreases the binding affinity of all the three inhibitors in clinical practice.

## Introduction

Production of β-lactamase enzymes is the commonest cause of bacterial resistance against β-lactam antibiotics. Beta-lactamases in Gram negative bacteria are responsible for the inactivation of β-lactam/β-lactamase inhibitor combinations. The increasing number of bacteria resistant to combinations of β-lactam and β-lactamase inhibitors challenges the ability to successfully treat serious urinary tract, respiratory tract, and bloodstream infections [1]–[3] and is creating difficulties in the treatment of serious hospital acquired infections. β-lactam antibiotics are known to be the most frequently prescribed antibacterial agents used to treat various infections in nosocomial and hospital settings. The continued introduction of newer β-lactam antibiotics and β-lactamase inhibitors to overcome β-lactam resistance has been driven by the increased number of β-lactamases including extended-spectrum (ESBL) and inhibitor-resistant phenotypes (IR). Currently, based on Ambler [4] classification scheme, β-lactamases are divided into four classes A to D. Class A, C, and D are serine β-lactamases (non-metallo), whereas class B enzymes are metallo-β-lactamases and need zinc ions in the active site for their action. β-Lactamases of Ambler's Class A enzymes are most frequently encountered in clinic isolates.

TEM and SHV are the most common mechanisms of bacterial resistance to β-lactam antibiotics are frequently reported in *Escherichia coli* and *Klebsiella pneumonia* [5], [6]. One of the most successful strategies used to overcome β-lactamase mediated resistance is the use of β-lactamase inhibitors [7]–[10]. Currently, three clinically used β-lactamase inhibitors (clavulanic acid, sulbactam and tazobactam) are in clinical use. In addition to these inhibitors, a number of other mechanism-based inactivators are also known for their inhibition profile. These mechanism-based inhibitors act by covalently modifying the active site of class A β-lactamases. However, the substitution of a single amino acid in the active site of β-lactamases reduces the affinity for β-lactamase inhibitors. Several previous studies observed the resistance pattern of inhibitor resistant TEMs (IRTs) and SHV-1 beta-lactamases. These studies help in explaining the cause of resistance to clavulanic acid and tazobactam [11]–[13]. The enzymes of SHV β-lactamase family has been heavily studied due to their importance in antibiotic as well as inhibitors resistance. Substitutions at ambler positions 130 were observed in clinically isolated SHV type betalactamases [14]–[15]. In the present study, we have focused on an inhibitor resistant point mutation (S130G) in SHV type class A β-lactamase. Mutation at this amino acid position (Ser130Gly), investigated in the present study is crucial for substrate and inhibitor specificity. Ser130Gly is the only point mutation which is responsible for inhibitor resistance in all the major class-A beta-lactamase (SHV, TEM and CTX-M) [16]–[19]. S130G mutation occurs in the highly conserved SDN loop of class A β-lactamases. Previous studies support the importance of Serine in the SDN loop (Ser130) as the base catalyst in substrate recognition and β-lactam ring opening [20]–[24]. Our study mainly involves the use of different *in silico* methods to investigate the affect of mutation (S130G) on the major physico-chemical properties in SHV type class A β-lactamase. We compared the dynamic behaviour of native and S130G mutant form of SHV β-lactamase by analyzing different properties like root mean square deviation (RMSD), H-bond, radius of gyration (Rg) and RMS fluctuation of mutation. The results clearly suggest that S130G mutation causes overall destabilization of the structure that may further lead to decrease in substrate specificity of SHV. The present study also focuses on the molecular docking analysis of clavulanic acid to investigate the detailed binding mechanism of S130G point mutant at molecular level. Comparison of this inhibitor resistant mutant to the wild type (WT) SHV will also allow us to gain better insights into some of the mechanisms evolved by the native enzyme to adopt inhibitor resistance profile and alterations in its biological function. Improved insights of structural and dynamic properties of SHV S130G mutants will provide a better insight and will be highly helpful in ameliorating the future drug designing approaches. Although many *in silico* studies have been reported in recent past on resistant mutants in SHV, to our knowledge this is the first study implementing molecular dynamics, docking and other *in silico* approaches to unravel the detailed mechanism of resistance due to S130G mutation in SHV. This study also provides an insight into the molecular mechanism of the phenotypic outcomes of S130G mutation, which includes the effects on stability, activity, binding and other properties.

## Material and Methods

### Molecular dynamic simulations

The crystal structures of wild type (PDB id 3D4F) and mutants (PDB id 1TDG) were used as starting structures for molecular dynamics simulations. The calculations were performed with GROMACS 4.5.5 package using the GROMOS 96 force field. The box dimensions ensured that any protein atom was at least 1.5 Å away from the wall of the box with periodic boundary conditions and solvated by simple point charge (spce) water molecules. NaCl counter ions were added to satisfy the electro-neutrality condition. Energy minimization was carried out using the steepest descent method. Berendsen temperature coupling and Parrinello-Rahman pressure coupling were used to keep the system in a stable environment (300 k, 1 bar), and the coupling constants were set to 0.1 and 2.0 ps for temperature and pressure, respectively. The partial mesh Ewald (PME) algorithm was employed for electrostatic and Van der Waals interactions; cut-off distance for the short-range VdW (rvdw) was set to 1.4 nm, where Coulomb cut-off (r coulomb) and neighbour list (rlist) were fixed at 0.9 nm. All the bond lengths were constrained using the LINCS algorithm, and the time step was set to 0.002 ps. The structures in a medium were equilibrated for 100 ps in NPT and NVT ensembles, respectively. Finally, a 10-ns molecular dynamics simulation was carried out for all the structures. Trajectories were stored every 2 ps. The RMSD (root-mean-square deviations) from the initial structure and the root-mean-square fluctuations (RMSF) were calculated during 10ns simulations. All the atoms of enzyme were included in the calculations of the root mean square fluctuations and radius of gyration.

### Molecular docking

Molecular docking analysis was carried out to identify the variations in the binding affinity of clavulanic acid with native and mutant structure of SHV retrieved at different time intervals (2 ns, 5 ns and 10 ns). GOLD (Genetic Optimization for Ligand Docking) 5.0 [25] was used for docking clavulanic acid against the native and mutant SHV at different time intervals. Docking annealing parameters for van der walls and hydrogen bonding were set to 5.0 and 2.5 respectively. The parameters used for genetic algorithm were population size 100, selection pressure 1.2, number of operations 1,00,000, number of islands 5, niche size 2, migrate 10, mutate 100 and cross -over 100.

### Bioinformatics analysis of the mutant

**Figure 1:**
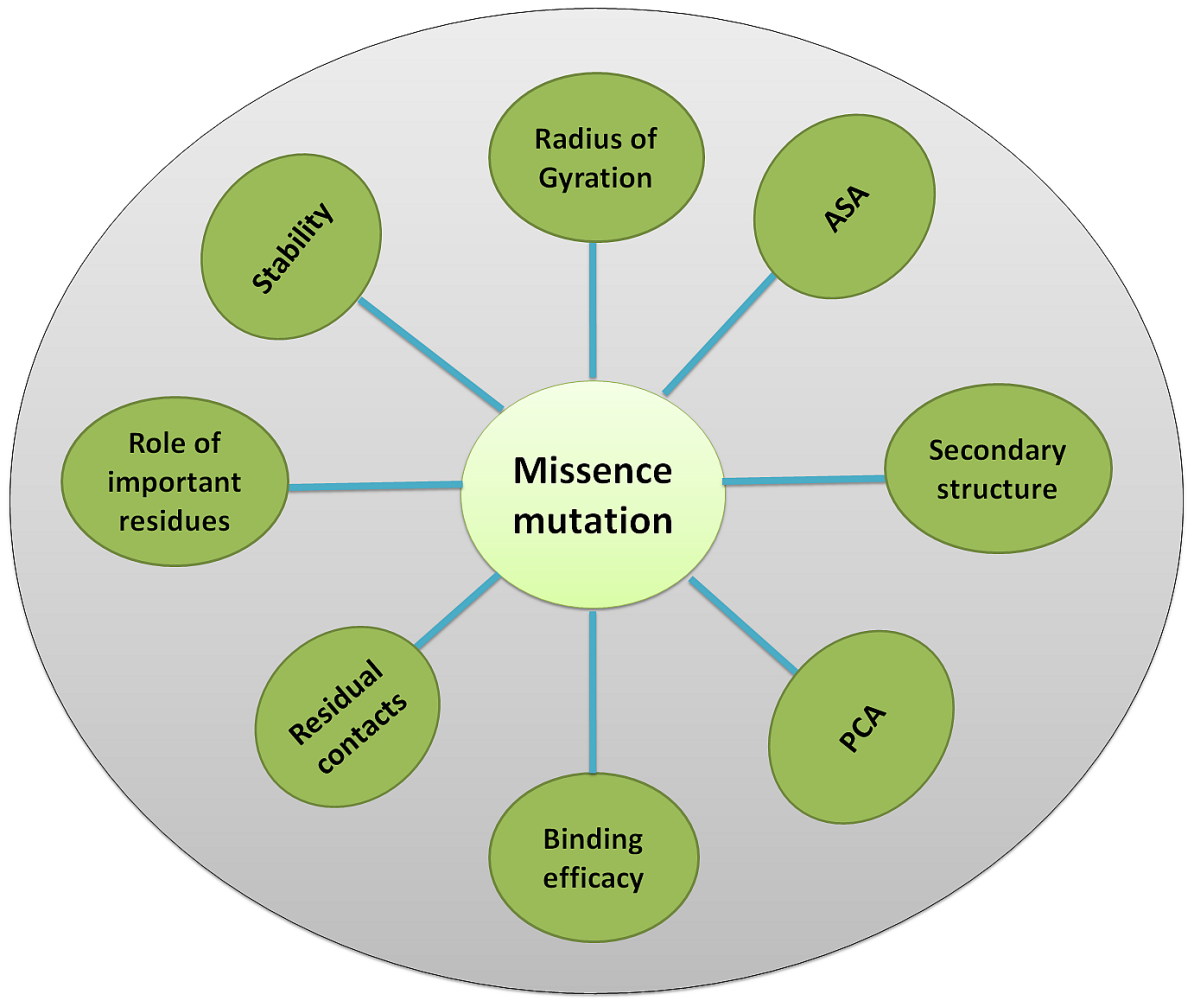
Physico-chemical features of native and S130G mutant SHV studied.

To examine the effect of point mutation on the change in protein stability, the difference in folding free energy (ΔΔG) was calculated by the CUPSAT [26], I-mutant-2, Mustrad and PoPMuSiC-2.0 program [27]. Pymol was used for the visual analysis of the enzymes. Ligplot [28] was used to analyze the interaction within the complex. (Fig 1)

## Results and Discussion

### Protein stability

In thermodynamics, the stability of an enzyme is a very important feature. Stability of an enzyme is a function of the free energy differences (ΔΔG) between the folded and unfolded state [29]. Predicting the stability of an enzyme using experimental as well as theoretical approaches has been an area of profound research over several years, [30]. CUPSAT server was used to investigate the effect of S130G mutation on the SHV-1 enzyme. The overall stability of the S130G mutant of SHV was calculated based on atom potentials and torsion angle potentials. The result revealed that SHV S130G was destabilized by the mutation effect which was probably due to the unfavorable torsion angles, and the atom potentials. All the point mutations responsible for inhibitor resistance in SHV were analyzed using CUPSAT. It was found that Ser130Gly and Ala187Thr were having destabilizing effect on SHV (Table 1). Ser130Gly was selected for further analysis because this mutation was within the active site of SHV β -lactamase and is the only point mutation which is responsible for inhibitor resistance in all the major class-A β -lactamases (SHV, TEM and CTX-M) [16]–[19]. The value of ΔΔG (predicted destabilizing energy) show that the mutant S130G (−2.73 kcal/mol) has significant influence on SHV-1 by the point mutation. A negative ΔΔG is an indication of protein destabilization, which is based on the change in stability of protein upon a point mutation by measuring the free energy difference between folded and unfolded state. Destabilization effect of S130G mutation was also observed through other programs too.

**Table 1:** Effect of point mutation on the protein structure and change in free energy.

Amino acid	Overall Stability	Torsion	Predicted ΔΔG (kcal/mol)
SER130GLY	Destabilising	Favourable	−2.73
LYS234ARG	Stabilising	Favourable	5.56
A187T	Destabilising	Unfavourable	−4.43
M9I	Stabilising	Unfavourable	1.73

Substitution of Serine to Glycine leads to change at the core of the protein that could result in the destabilization of the loop.

### Effect on hydrogen bond network

**Figure 2:**
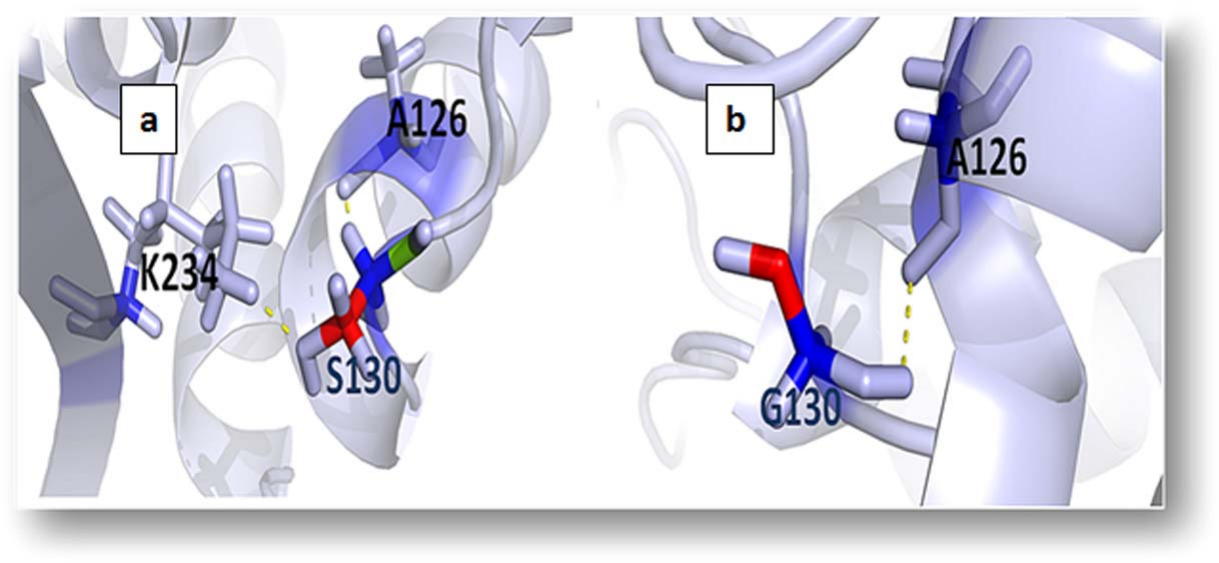
(a) the wild type with SHV in the left panel and its neighbors in the wild type are shown (b) the S130G mutant in the right panel and its neighbors in the mutant type its neighbors in the wild type are shown.

Hydrogen bonds, which are considered to be the key constituents of biomolecular structures [31], participate in the formation of secondary [32], tertiary and quaternary structures [33] of proteins. The drift in structural property of S130G can result in loss of hydrogen bonds and disturb correct folding and could have a significant impact on the structural integrity. This mutation also cause disturbance in the interactions with other molecules or other parts of the protein. Figure 2a illustrates the distance between native S130, neighboring residues A126 and K234. The native S130 maintains the distance range of 2.9 Å −3.2 Å between neighboring residues K234 and A126 respectively, while in mutant (S130G) substitution of glycine, the distance between mutant G130 and its neighboring residue, A126 (distance 3.3 Å) (Figure 2b). Mutation results in the changes in local environment of SHV and enhance in the modification of residues distances. As can be seen from figure, the wt residue, S130, is involved in several hydrogen bonds, which are absent in the mutant.

### Molecular dynamics

#### RMSD

**Figure 3:**
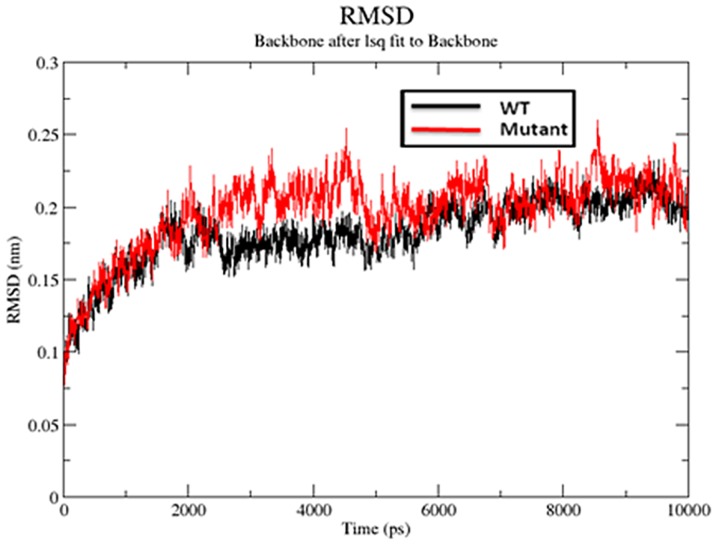
Backbone RMSDs are shown for native and S130 mutant SHV β-lactamase at 300 K, black color indicate native SHV, point mutant form S130G SHV β-lactamase shown in red.

In the present study, comparative molecular dynamics simulations were performed in order to study the difference in protein dynamics of wild type and its mutant. The main chain root mean square deviations were calculated for the trajectories of the wild and mutated SHV. The RMSD was calculated using the trajectory file for backbone after least square fit to C-alpha using “g_rms” tool. It is evident that the wt (3D4F) and mutant structure (1TDG) remain close at their starting conformation till 2000 ps which results in a backbone RMSD of about ∼0.2 nm (Figure 3). Between ranges of 2000–5000 ps, structure of SHV wild type attained a maximum RMSD value of ∼0.23 nm while the S130G mutant attained a highest deviation of ∼0.27 nm. From 5000 ps till 9000 ps, a slight deviation in RMSD was also observed, where mutant S130G retained a large deviation from wt, with a maximum RMSD of ∼0.27 nm around 9000 ps. The RMSD of both the structures (wt and mutant) remained constant throughout the rest of time period.

**Figure 4:**
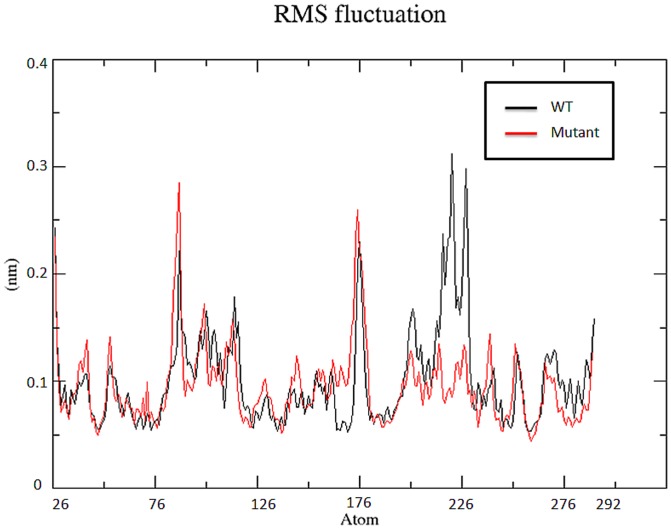
RMSF of the backbone atoms of native and mutant SHV β-lactamase vs. time at 300 K. Native is shown in black; S130G point mutant form of SHV β-lactamases is shown in red.

The backbone RMSD graph suggests that the wild type protein is more stable than S130G mutant. Small variation in the average RMSD values of wt and mutant lead to the conclusion that this mutation could affect the dynamic behavior of SHV, thus providing a suitable basis for further analyses. For determining the effect of mutation on dynamic behavior of residues, RMSF values of mutant and native structure were calculated. RMSF value of native residues fluctuates from a range of ∼0.08–0.32 nm in the entire simulation period. Moreover, mutant S130G exhibited a maximum flexibility of ∼0.29 nm (Figure 4). Analysis of the fluctuations revealed that the greatest degree of flexibility was shown by both the native as well as mutant model. The main chain root mean square fluctuations calculated over the trajectories and averaged over each residue for the wild-type and mutant during the molecular dynamics simulations shows that fluctuations of amino acids were largest in the region 90–105 and 200 to 240. Slight fluctuation was also observed at residues 128–131 for wt and mutant enzymes (Figure 4). Amino acids falling within this region show strong binding (hydrogen bonds and hydrophobic contacts) as reported in our previous [34], [5] as well as others studies. Moreover, the RMSF value for important loops (SDN and KTG) for substrate or drug binding remains low throughout the molecular dynamics simulation (Figure 4). The outcome of this is in great agreement with previous studies which shows that these loops play an important role in substrate or drug binding [7]. Figure 3 shows the RMSD of the wt and mutant SHV superimposed in the same scale.

#### Potential energy

**Figure 5:**
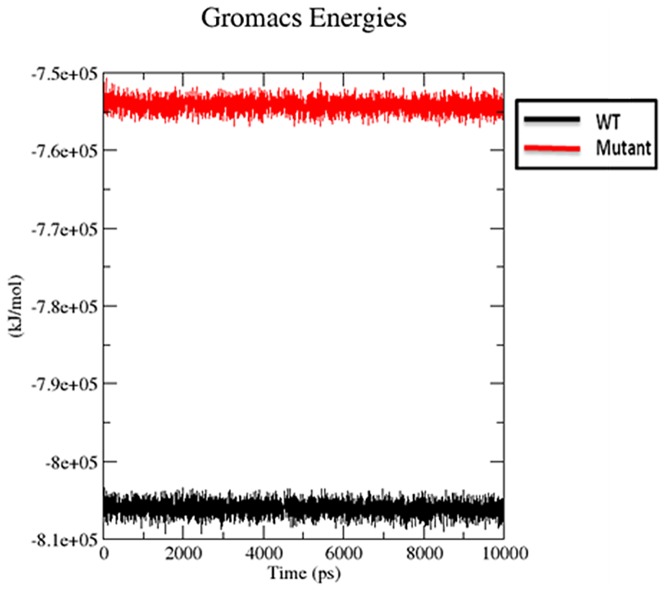
Potential energy (kJ/mol) of native and mutant type SHV β-lactamases. Native is shown in black; S130G mutant form of SHV β-lactamases is shown in red.

The potential energy analyzed during the 10 ns molecular dynamics simulations for wild type and the mutant are shown in figure 5. The potential energy plot shows that all the molecular systems stabilized and remained stable throughout 10 ns of molecular dynamics simulations. The average of potential energy for each MD simulation shows that the potential energy during MDs with the wild type stabilized at approximately −806049.3047 kcal/mol, while the potential energy of mutant was found to be stabilized at approximately −754239.1479 kcal/mol.

#### Radius of gyration and SASA

**Figure 6:**
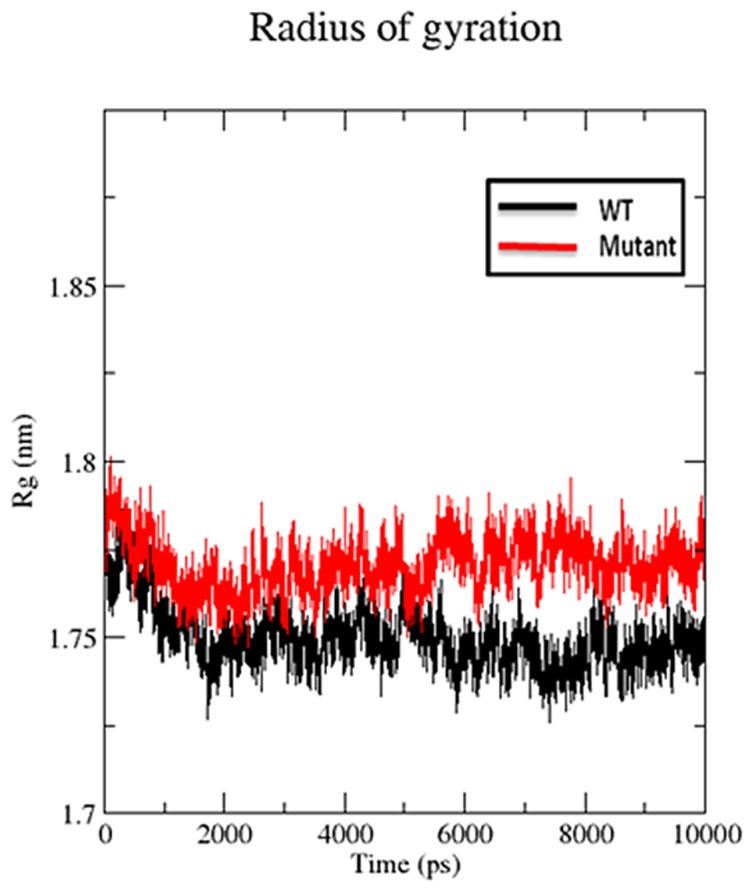
Rg of Cα atoms of native and mutant type SHV β-lactamases. Native is shown in black; S130G mutant form of SHV β-lactamases is shown in red.

**Figure 7:**
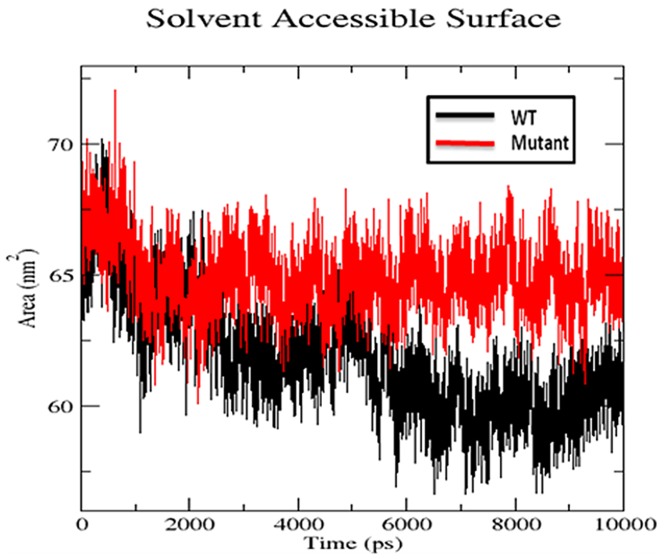
Solvent accessible surface area of native and S130G mutant of SHV β-lactamases. Native is shown in black; S130G mutant form of SHV type β-lactamase is shown in red.

We also used radius of gyration and solvent accessible surface area analysis to determine compactioness level in the structure and solvent accessibility of native and S130G mutant SHV. Radius of gyration (Rg) is defined as the mass weighted root mean square distance of a collection of atoms from their common center of mass. Analysis of radius of gyration provides us an insight of the overall dimensions of the protein. Figure 6 illustrates the plot of Rg of Cα atoms of the wt and S130 mutant of SHV for 10 ns simulation time at 300 K. The radius of gyration (Rg) of wild type and S130G mutant was found to be 1.73 and 1.77 nm respectively. The curve reveals that the mutant shows higher Rg value than SHV WT during the whole simulation time period. From the Rg graph, it is revealed that S130G mutation with SHV decreases the stability of the enzyme. Figure 7 shows the change of SASA of native and S130G mutant of SHV with time. SASA plot indicates greater value of SASA for S130G mutant with time as compared to its wt counterpart. From radius of gyration (Rg) plot, it was revealed that, the stability of the S130G SHV mutant is slightly reduced. The results of SASA analaysis was found to be supported by Rg plot [35].

#### H-bond analysis

**Figure 8:**
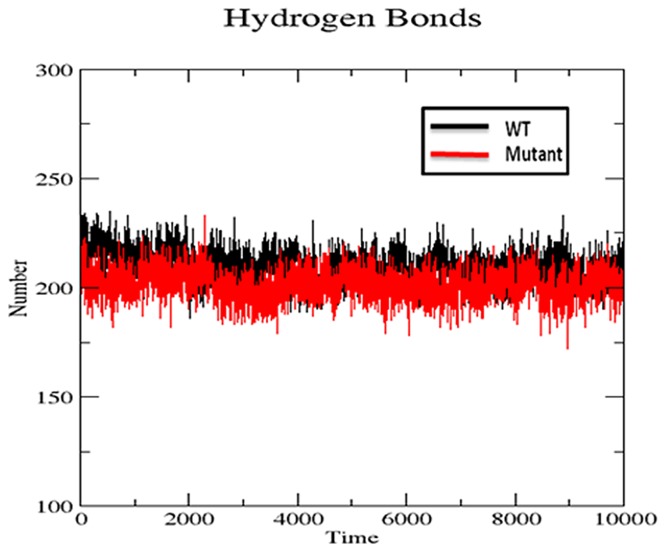
Average numbers of protein-solvent intermolecular H-bonds in WT and S130G mutant type of SHV β-lactamase. SHV wt is shown in black; S130G point mutant form of SHV is shown in red.

Hydrogen bonds are considered to be playing vital role in molecular recognition and the overall stability of the protein structure [36]. H-bond analysis was performed to analyze the intermolecular H-bond for native and S130G SHV mutant structure during the 10ns simulation period. Figure 8 shows the difference in protein–solvent interactions in native and mutant SHV's structure. The number of H-bond was found to decrease in S130G mutant of SHV. It was revealed, that more intermolecular H-bonds within native SHV were very helpful to maintain its rigidity. The native structure of SHV was more rigid and also showed higher participation in H-bonding with other amino acids. From RMSF plot (Figure 4) and H-bond analysis of both the structures, it was observed that S130G mutation in SHV led to high degree of conformation flexibility due to lesser number of hydrogen bonds formation. The binding stability of mutant was also found to be effected because of less number of H-bonds formation during the whole simulation time. This was is great agreement with the previous study, which illustrates that the active site mutation in class-A β-lactamase lowers the H-bond form [37].

#### Eigenvectors

**Figure 9:**
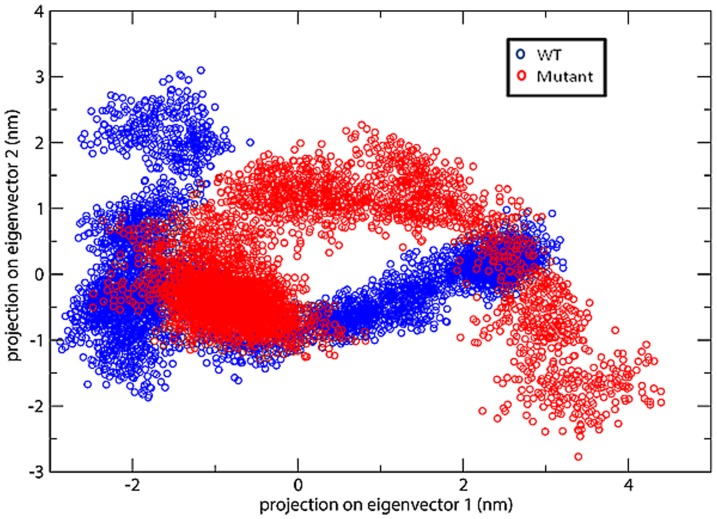
Projection of most significant principal components of motion of the Cα-atoms of native and mutant SHV β-lactamase. The trajectory projected to the two-dimensional space. Native is shown in blue; S130G point mutant form of SHV is shown in red.

Eigenvectors, a Principal component analysis (PCA) method was further used in order to determine the overall strenuous motions within both wt and mutant structure of SHV. Figure 9 projects the eigenvector graph for 10 ns, where the snapshots were taken at every 2 ps. The eigenvectors of the covariance matrix are called its principal components. The trajectories of eigenvector graph projects the structural motion of wt and mutant SHV in phase space, where eigenvalues represents the structural motion. Figure 9 illustrates the 2D plots of principal components for native and S130G mutant form of SHV type β-lactamase. The distribution of dots within the graph indicates level of conformational changes within the protein structure. It was found that the internal motions of the S130G mutant structure are much bigger than the SHV wt. From this study it could be revealed that the strenuous motions increased in S130G mutant and is in agreement with MD analysis.

### Secondary structure

**Figure 10:**
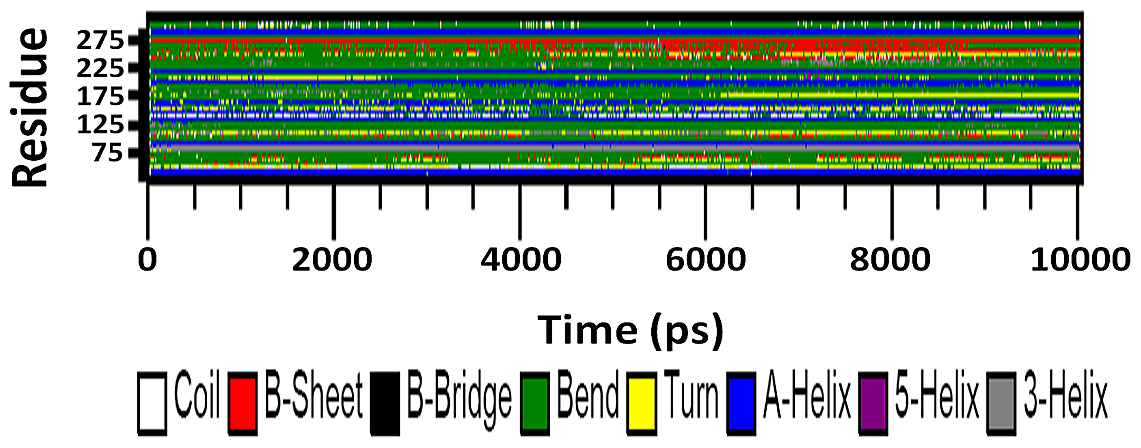
Time evolution of the secondary structure elements of SHV wt at 300 K. The color scale at the bottom represents the DSSP classification of each secondary structure element.

**Figure 11:**
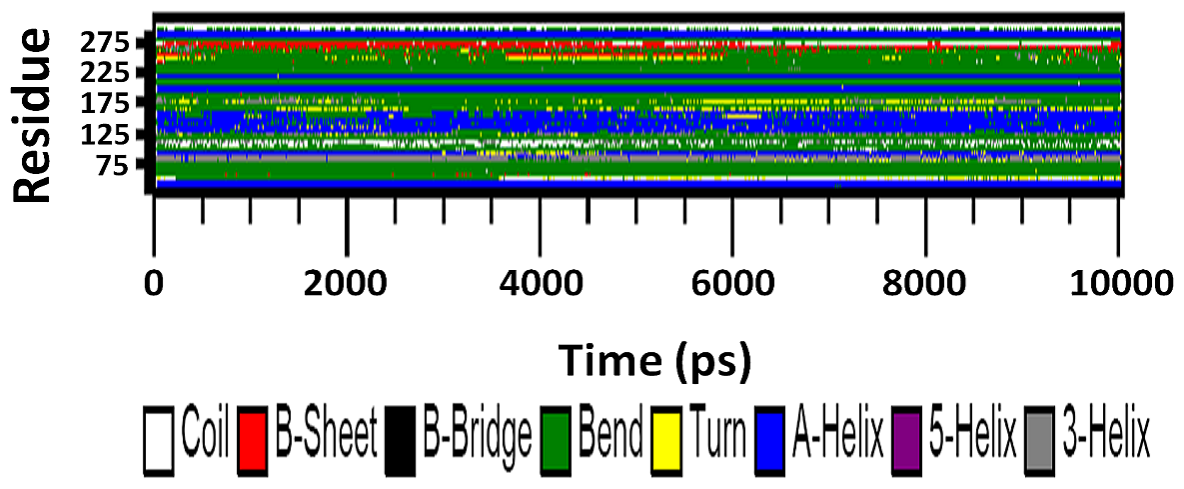
Time evolution of the secondary structure elements of S130G mutant of SHV at 300 K. The color scale at the bottom represents the DSSP classification of each secondary structure element.

Secondary structure elements were also observed to monitor the stability of the various simulations. Figures 10 and 11 shows the secondary structure elements of the wild type and mutant SHV during 10 ns simulations. This study reveals that most populated conformations of native SHV structure preserved its secondary structural elements and was maintained for the remaining clusters during the whole time period as well. Unlike the SHV native structure, the secondary structural elements of S130G were found to be distorted. The mutated residue (G130) showed a significant variation in secondary structural elements compared to the same position in the native form (S130). It was observed that S130G mutant structure significantly differs from the wt structure, which was in a great agreement with the RMSD and RMSF plots. It was further revealed that the surrounding residues of mutation also show modification in all the clusters. Taken together, it was evident that S130G mutation causes major structural changes in the overall structure further destabilizes the protein.

### Molecular docking

**Figure 12:**
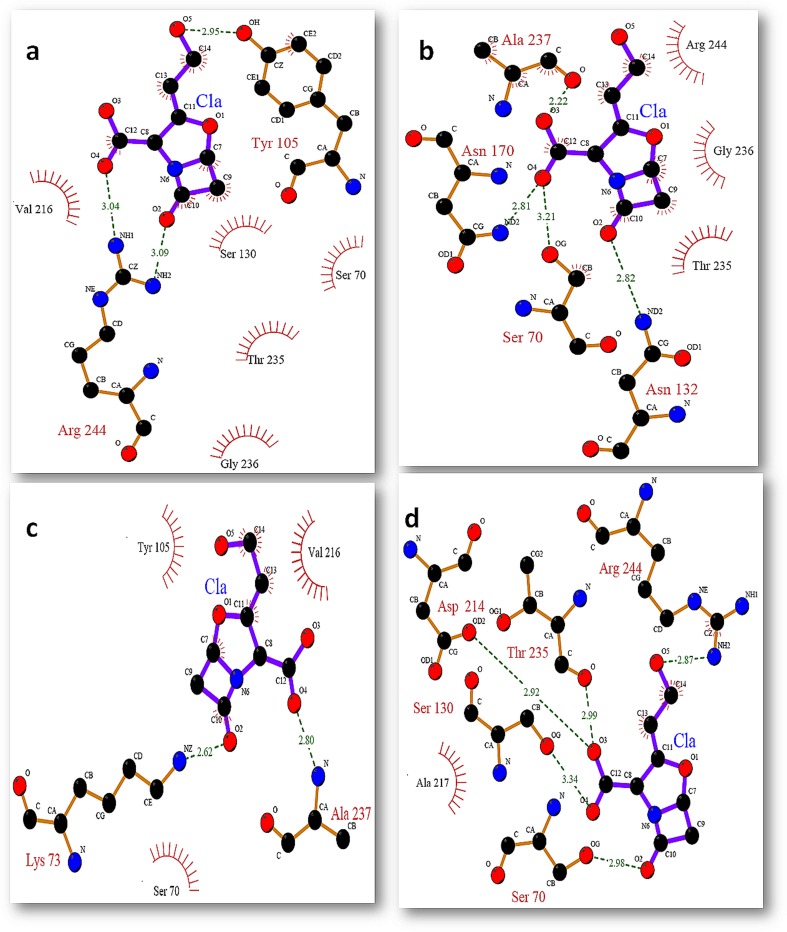
Binding mode of clavulanic acid with active site residues of SHV wt at different time intervals (a) binding of ca at 0 ns (b) binding of ca at 2 ns (c) binding of ca at 5 ns (d) binding of ca at 10 ns.

**Figure 13:**
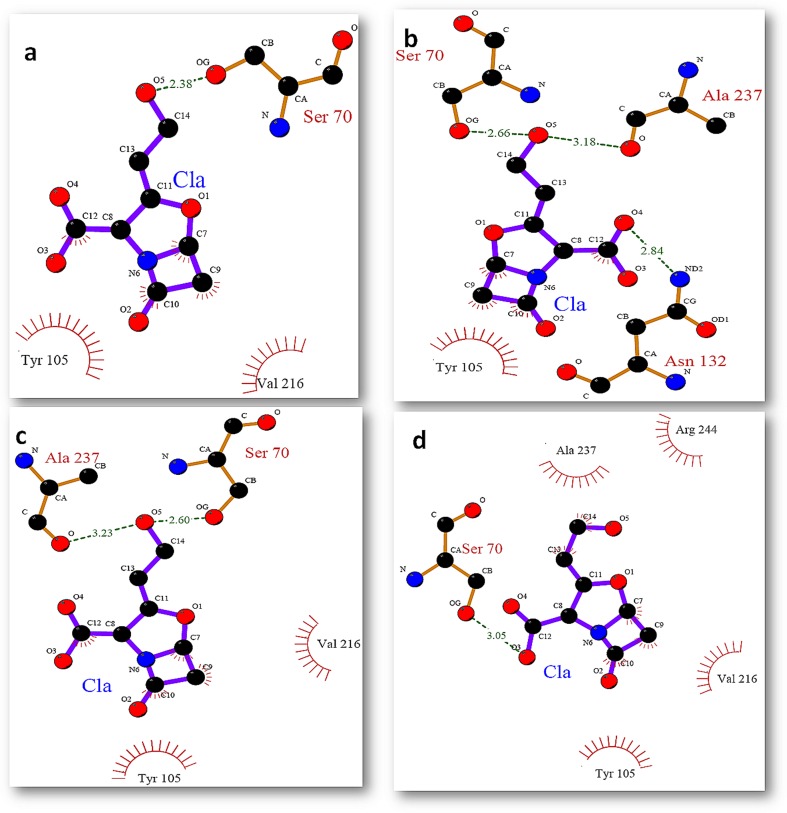
Binding mode of clavulanic acid with active site residues of SHV S130G mutant at different time intervals (a) binding of ca at 0 ns (b) binding of ca at 2 ns (c) binding of ca at 5 ns (d) binding of ca at 10 ns.

In this study we used molecular docking approach to inspect the effect of mutation on the binding of clavulanic acid within the active site of native and mutant SHV at different time intervals. It was revealed that S130G mutation has a great impact on the binding. The inhibitor used in the present study was not found to bind effectively within the binding site of S130G mutant. All the docking experiment was performed in the presence of active site water molecules. This study illustrates that clavulanic acid was unable to bind within the active site of mutant as compare to its wild type. Figures 12 and 13 show the binding of clavulanic acid within the active site of wt and mutant SHV at different time intervals. It has been clearly illustrated that the binding pocket of SHV wt was accommodating the inhibitors in a very well manner by making a number of H-bonds and hydrophobic contacts with the active site residues as compare to S130G mutant, where the binding of clavulanic acid was found to be very weak. It was observed that in S130G mutant, S70 was making H-bond with clavulanic acid at all instances.

This was because of the improper binding of clavulanic acid within the active site of mutant, that the binding efficacy of the inhibitor was very much lower as compare to its wt.

The binding poses for clavulanic acid against the native and mutant structures of SHV at different time intervals were determined and different poses were further generated based on the goldfitness score of each pose. Gold fitness score is an indicator of the binding affinity of a ligand–receptor complex. The goldfitness scores for clavulanic acid against both the native as well as mutant SHV are depicted in Tables 2 and 3. The binding affinity, hydrogen bond, and hydrophobic interaction of clavulanic acid with native and mutant form of SHV type β- lactamase at different time intervals are analyzed and the detail of binding residues are given in Tables 1–2. On comparing the binding affinity of clavulanic acid, it was found that this β-lactamase inhibitor show low docking score with mutant SHV type β-lactamase (Tables 1 and 2). From the docking results, the variation in the binding affinity of clavulanic acid with native and mutant structure of SHV at different time intervals is observed. Clavulanic acid was found to show high binding affinity with the native SHV type β-lactamase where as for S130G mutant, binding affinity of this β-lactamase inhibitor is decreased. The binding mechanism of clavulanic acid with native and mutant SHV type β-lactamase is further investigated in detail. Binding residues are analyzed using Ligplot [28]. Figures 12 and 13 show the binding of clavulanic acid within the active site of WT and mutant SHV at different time intervals. Clavulanic acid that has been reported to be an effective inhibitor for Class-A β-lactamase was found to bind within the active site of wt with higher efficacy; this supports the role of hydrogen bond and hydrophobic interactions and the efficacy of clavulanic acid against wt enzyme. It was observed that the mutated Glycine residue at 130th position was mostly involved in making hydrophobic contacts with the selected inhibitors. Along with G130 other residues were also found to be playing important role in the binding of inhibitor within the active site of SHV S130G mutant. K-73 was found to be a key residue that was actively involved in hydrogen bond formation at several instances. Apart from K-73 there were some other residues that were actively involved in the positioning of inhibitor within the active site they include S70, N132, G130, Y105 and V216. G130 that was the mutant residue was found to be making only hydrophobic contacts with the inhibitors.

**Table 2:** Binding efficacy of clavulanic against SHV wt at different time intervals and the active site residues involved in the binding.

Time period	Gold fitness score	Residues	
		Hydrogen Bond	Hydrophobic interaction
**0 ns**	33.81	Y105, R244	S70, Y105, S130, V216, T235, G236
**2 ns**	35.25	S70, N132, N170, A237	S70, T235, G236, A237, R244
**5 ns**	37.03	K73, A237	S70, Y105, V216
**10 ns**	36.05	S70, S130, D214, T235, R244	A237, R244

**Table 3:** Binding efficacy of clavulanic against SHV S130G mutant at different time intervals and the active site residues involved in the binding.

Time period	Gold fitness score	Residues	
		Hydrogen Bond	Hydrophobic interaction
**0 ns**	28.24	S70	Y105, V216
**2 ns**	31.27	S70, N132, A237	Y105
**5 ns**	36.67	S70, A237	Y105, V216
**10 ns**	26.27	S70	Y105, V216, A237, R244

### Mutant-stability upon binding of clavulanic acid

The difference in the hydrogen bond for inhibitor with protein is shown in docking analysis (ligplot). We also calculated the stability of the protein to analyze the inhibitor-specific effect of S130G mutation. Total energy of both the wild type and S130G were calculated and was found to be −49.0909 Kcal/mol and −24.3778 Kcal/mol respectively. The solvent accessible surface area of the active site of both wild and mutant protein was also calculated, which also shows slight difference in the surface of mutant (2229.02) compare to its wild type (2402.93).These stability calculation also proves that mutant destabilize the protein compare to wild type.

## Conclusion

Overall, the present study on the structural consequences of S130G point mutant form of SHV type class A β-lactamases showed the contribution of this important and critical amino acid residue in the biological functions. Further, the conformational changes of SHV were analyzed. This study revealed that the conformational flexibility of the S130G point mutant significantly increases as compared to the native wild type SHV structure. Other structural characteristic features like RMSD, RMSF, Rg, secondary structure, SASA, H-bond, and PCA plots revealed that S130G mutation in SHV lead to flexible conformation and also affect the binding behaviour of mutant. Further, molecular docking simulation performed to investigate the binding affinity of clavulanic acid with native and mutant SHV. The result revealed decrease in substrate specificity of SHV due to S130G mutation. The H-bonds during entire simulation differed between the native and mutant SHV, the decreased number of H-bonds may affect the binding stability. Therefore, this study demonstrated that the S130G point mutation in SHV type class A β-lactamases confer many structural as well as chemical changes within SHV, which further leads to inhibitor resistance. Improved insights of structural and dynamic properties of SHV S130G mutants will be highly helpful in ameliorating the future drug designing approaches. Although many experimental studies have been reported in recent past on resistant mutants in SHV, to the best of our knowledge this is the first study where molecular dynamics, docking and other *in silico* studies are used to unravel the detailed mechanism of resistance due to S130G mutation in SHV. Additionally this study also provided an insight into the molecular mechanism of the phenotypic outcomes of mutations, which includes the effects on stability, activity, binding and other properties.
